# Amino acid metabolism in glioma: in vivo MR-spectroscopic detection of alanine as a potential biomarker of poor survival in glioma patients

**DOI:** 10.1007/s11060-024-04803-2

**Published:** 2024-08-27

**Authors:** Seyma Alcicek, Ulrich Pilatus, Andrei Manzhurtsev, Katharina J. Weber, Michael W. Ronellenfitsch, Joachim P. Steinbach, Elke Hattingen, Katharina J. Wenger

**Affiliations:** 1 Goethe University Frankfurt, University Hospital, Institute of Neuroradiology, Schleusenweg 2-16, 60528 Frankfurt/Main, Germany; 2 University Cancer Center Frankfurt (UCT), Frankfurt/Main, Germany; 3 Frankfurt Cancer Institute (FCI), Frankfurt/Main, Germany; 4 German Cancer Research Center (DKFZ) Heidelberg, German Cancer Consortium (DKTK), Partner Site, Frankfurt/Mainz, Germany; 5 Goethe University Frankfurt, University Hospital, Institute of Neurology (Edinger-Institute), Frankfurt/Main, Germany; 6 Goethe University Frankfurt, University Hospital, Dr. Senckenberg Institute of Neurooncology, Frankfurt/Main, Germany

**Keywords:** Gliomas, Amino acid metabolism, Alanine, Magnetic resonance spectroscopy, Overall survival, IDH mutation

## Abstract

**Purpose:**

Reprogramming of amino acid metabolism is relevant for initiating and fueling tumor formation and growth. Therefore, there has been growing interest in anticancer therapies targeting amino acid metabolism. While developing personalized therapeutic approaches to glioma, in vivo proton magnetic resonance spectroscopy (MRS) is a valuable tool for non-invasive monitoring of tumor metabolism. Here, we evaluated MRS-detected brain amino acids and myo-inositol as potential diagnostic and prognostic biomarkers in glioma.

**Method:**

We measured alanine, glycine, glutamate, glutamine, and myo-inositol in 38 patients with MRI-suspected glioma using short and long echo-time single-voxel PRESS MRS sequences. The detectability of alanine, glycine, and myo-inositol and the (glutamate + glutamine)/total creatine ratio were evaluated against the patients’ IDH mutation status, CNS WHO grade, and overall survival.

**Results:**

While the detection of alanine and non-detection of myo-inositol significantly correlated with IDH wildtype (*p* = 0.0008, *p* = 0.007, respectively) and WHO grade 4 (*p* = 0.01, *p* = 0.04, respectively), glycine detection was not significantly associated with either. The ratio of (glutamate + glutamine)/total creatine was significantly higher in WHO grade 4 than in 2 and 3. We found that the overall survival was significantly shorter in glioma patients with alanine detection (*p* = 0.00002).

**Conclusion:**

Focusing on amino acids in MRS can improve its diagnostic and prognostic value in glioma. Alanine, which is visible at long TE even in the presence of lipids, could be a relevant indicator for overall survival.

**Supplementary Information:**

The online version contains supplementary material available at 10.1007/s11060-024-04803-2.

## Introduction

Diffuse gliomas are the most common primary malignant brain tumors in adults. Their aggressive variants are associated with a lack of effective treatment strategies, high recurrence and mortality rates, and a short overall survival [1, 2]. The fifth edition of the World Health Organization (WHO) Classification of Tumors of the Central Nervous System (CNS), published in 2021, has introduced major changes that advance the role of molecular diagnostics in CNS tumor classification [3]. Some entities are now readily and consistently characterized by defining molecular features such as the isocitrate dehydrogenase (IDH) mutation status. The final, integrated diagnosis combines tissue-based histological and molecular diagnosis and is a first step towards precision medicine [4, 5]. More recent advances in classification included incorporation of metabolism alongside therapeutic vulnerabilities in glioblastomas [6, 7]. Metabolic changes in gliomas generally result from the interplay between cancer cells and the cells in the microenvironment. These interactions form a complex system with a large number of simultaneous reactions across multiple compartments, and a flexible network in which different metabolic pathways use the same compounds [8, 9]. One important metabolic module is the reprogramming of amino acid metabolism (i.e., alterations in the rate of amino acid uptake, amino acid metabolic pathways, amino acid levels, or key metabolic enzymes in tumor cells) facilitating glioma proliferation, immune escape, and chemoresistance. Amino acid pool could serve as potential markers for diagnosis, prognosis, and as additional therapeutic targets [10–13].

In the course of developing therapies targeting altered tumor metabolism, proton magnetic resonance spectroscopy (^1^H MRS) has proven to be a valuable tool as it enables non-invasive in vivo observation of tissue metabolism by exploiting the magnetic properties of atomic nuclei in cell metabolites [14, 15]. This technique provides the unique opportunity to collect metabolic fingerprints of tumor manifestations in patients with the ultimate goal of identifying markers for therapy stratification, monitoring treatment response, prediction, and prognosis [16]. Over the decades, the most prominent standard singlet MR signals of choline (Cho), creatine (Cr), N-acetyl aspartate (NAA) are employed in ^1^H MRS for tumor diagnosis and prediction of treatment as well as overall survival [17]. However, the MRS detection of amino acids, which may provide more detailed information on altered tumor metabolism [12, 18], is hampered by their low concentration and overlapping complex signal patterns which are modulated under J-coupling evolution. Since alanine (Ala) (~ 0.5 mM) [19] and glycine (Gly) (~ 0.6 mM) [20] concentrations in a healthy brain are low, these metabolites are commonly not detected using standard MRS techniques. But, in particular glioma subtypes (i.e., where there is prominent reprogramming of amino acid metabolism), the concentrations of these amino acids could increase, and they can become evident in vivo MR spectra. On the other hand, while myo-inositol (mIns) (~ 4 mM) is detectable in a healthy brain using in vivo MRS, in specific glioma subtypes, the level of mIns can decrease below the MRS concentration limit [20].

In this study, we investigated amino acids Ala, Gly, glutamate (Glu), and glutamine (Gln) as potential biomarkers for glioma-defining molecular features (i.e., IDH mutation status), CNS WHO grade and patient overall survival. To overcome the inherent problems of overlapping signals, we recorded in vivo data at short and long echo times (TEs). For the reason explained above, the rather conservative approach presented here resorts to a binary quantification with two categories: detectable/non-detectable except for Glu and Gln as their sum (Glx) can be quantified with higher spectral fitting precision. As the detection of Gly interferes with mIns in tumor tissue due to signal overlap, we also report mIns, which is a marker of astrogliosis, and its high level might be related to increased membrane turnover or damage to myelin sheets [21, 22].

## Materials and methods

### Clinical study design

38 patients with mostly untreated WHO II–IV gliomas (at the time of study initiation according to WHO 2007 classification of tumors of the central nervous system [23]) were enrolled in the study, prospectively. The study protocol was approved by the institutional review board (Ethics Committee, University Hospital Frankfurt, Germany, project No: SIN-04–2014) and written informed consent was obtained from the patients. IDH mutation status was determined by immunostaining (Anti-IDH1 R132H antibody), Infinium Human Methylation 450 BeadChip analysis, and/or DNA sequencing. Cases were reclassified into the 2021 WHO classification of tumors of the CNS for this report.

### Clinical MR study protocol

Patients were scanned using an MRS protocol on a clinical whole-body 3T MR Scanner (Magnetom Trio, Siemens Healthineers, Erlangen, Germany) with a double-tuned ^1^H/^31^P volume head coil (RAPID Biomedical GmbH, Rimpar, Germany). The protocol included T2-weighted imaging (T2WI) turbo spin-echo (TSE) in a transverse plane, 3D T1-weighted imaging (T1WI) gradient echo, and two ^1^H single-voxel spectroscopy (SVS) point-resolved spectroscopy (PRESS) measurements at TE = 30 ms and TE = 97 ms with optimized echo spacing for detection of 2-hydroxyglutarate (2-HG) [24] from the tumor area defined on T2WI TSE. SVS acquisitions were performed for volumes of 8 mL (20 × 20 × 20 mm) from identical target positions with the same B0 shimming parameters, covering the solid part tumor and peritumoral tissue. Table 1 provides a detailed MRS sequence protocol (See Supplementary Information for MRSinMRS Reporting Checklist).

**Table 1:** MRS sequence protocol. During the session, 2D T2WI TSE in the axial plane, a 3D T1WI gradient echo, and ^1^H SVS PRESS with two different TE were recorded using a double-tuned ^1^H/^31^P volume head coil. SVS, single voxel spectroscopy; PRESS, point resolved spectroscopy

Pulse sequence	^1^H SVS PRESS	^1^H SVS PRESS
TE	30 ms	97 ms
TR	3000 ms	3000 ms
Flip angle	90°	90°
Voxel size	20 × 20 × 20 mm^3^	20 × 20 × 20 mm^3^
Vector size	1024	1024
Bandwidth	1200 Hz	1000 Hz
Acquisition time	5:00 m	6:36 m
Number of Averages	96	128

### Spectral analysis

LCModel [25] was used for the ^1^H spectral analysis. A 3D volume-localized basis set was simulated for SVS at TE = 30 ms and TE = 97 ms using NMRScopeB which is implemented in jMRUI (Version 5.2, available at http://www.mrui.uab.es*).* The simulation assumed a 20 × 20 × 20 mm^3^ volume, homogeneously filled with the respective metabolites. The basis set spectra included 2-HG, NAA, Glu, Cr, Gln, Cho, mIns, lactate (Lac), and Ala. ^1^H spectra were analyzed with and without the Gly singlet peak simulation implemented in LCModel. The spline function in LCModel which models a baseline composed of macromolecules and lipid signals was used applying the control parameter DKNTMN (minimum allowed spacing between spline knots) of 0.15 ppm. The phased, fitted, and residual spectra from the LCModel analysis were evaluated to check the quality of the fit. The following rejection criteria were applied: existing artifacts, metabolite linewidth (FWHM) > 0.1 ppm, and signal-to-noise ratio < 3. To determine the spectral fitting quality, rejection thresholds were defined based on Cramer-Rao lower bounds (CRLBs) given by LCModel fit results, which are CRLB < 10% for total Cho and total Cr (tCr), < 15% for Glu + Gln (Glx). In this study, we focused on the amino acids Gly and Ala, which in healthy brains are below the detection limit but their concentration can be increased for different tumor subgroups. A detection limit in MRS can be determined by giving a maximum value for the CRLBs. According to the Experts’ Consensus Recommendations, *“a high relative CRLB may indicate that a metabolite is not detectable in an individual spectrum*,* which may be biologically significant when a diagnostic decision needs to be made with a single spectrum”* [26]. Therefore, in this study, when spectral quality parameters for the standard MRS metabolites were matched, and CRLB values were < 40% for mIns, Ala, and Gly, metabolite concentrations were assigned to (mostly) low but detectable concentrations of the respective metabolite, and its ratio to tCr (mean ± standard deviation) was reported. For patients, where spectral fitting for metabolites of interest resulted in CRLB values higher than defined thresholds, i.e., fitting uncertainty was higher than expected for reliable quantification of the metabolite as a result of its low concentration, the metabolites were assigned as non-detectable (i.e., concentration is lower than detection limit for reliable metabolite quantification). In a nutshell, we consider that relative CRLB could be used as an indirect measure of the estimated metabolite concentration if spectral/fitting quality measures are consistent for all MRS data in the study. Further, the detectability of Ala was also visually validated in each spectrum due to its position close to the Lac and lipid signals. CRLB thresholds were defined taking into account the low concentrations of mIns, Ala and Gly reported in the literature [27]. With this binary approach, we follow the recommendation not to exclude data from statistical analysis based on CRLB values for low-concentration metabolites [28]. We instead used these values to determine the limit of detectability for the given standard MRS protocol. CRLB values and metabolite intensities given by LCModel for Ala, Gly, and mIns are presented in **Supp** Fig. 1. In the initial study design, PRESS at TE = 97 ms was primarily aimed at 2-HG detection [29–31]. Due to the near absence of lipids and macromolecules in the Ala spectral region and the inverted spectral pattern of Ala in the spectra acquired using TE of 97 ms, it was used for Ala quantitation. For mIns and Glx quantitation, the short TE was chosen as an optimal detection time to avoid signal loss induced by T2 decay and dephasing due to strong couplings. As suggested by Tiwari et al [12], Gly was quantified using spectra acquired at TE of 97 ms. Because of J-evolution, the intensity of the mIns peak signal at 3.55 ppm, which overlaps with the singlet of Gly at 3.55 ppm, decreases approximately four times by increasing the TE from 30 ms to 97 ms, resulting in a pattern with positive and negative signals (see Results section for the spectral pattern). While at long TE (97 ms), the mIns signal at 3.64 ppm facilitates the distinguished fitting of mIns and Gly, short TE, where larger signals are obtained, is favorable for mIns quantification. Metabolite signal ratios were corrected for T_1_ and T_2_ relaxation using previously calculated relaxation times [32]. Registration of the SVS data to 3D-anatomical data was performed using Gannet software (version 3.3.1) [33] and SPM12 (version 7771) [34].

### Statistical analysis

The statistical analysis was performed using the OriginPro software (version 2020; OriginLab Corp., Northampton, MA, USA). Due to the non-normal distribution of spectral quality parameters, assessed using a Shapiro-Wilk test, a Kruskal-Wallis test (nonparametric version of classical one-way analysis of variance, ANOVA) was used to compare the spectral quality parameters (i.e., FWHM and SNR) between groups. A chi-square test was performed to compare the detectability of metabolites (i.e., Ala, Gly, and mIns) in tumor spectra acquired at short and long TE. Because of the normal distribution of the Glx/tCr ratio, assessed using a Shapiro-Wilk test, a one-way ANOVA with Tukey’s post-hoc analysis was used to compare the Glx/tCr ratio between tumor grades and a two-sample t-test to compare the Glx/tCr ratio in IDH-mutated (IDHmut) to IDH*-*wildtype (IDHwt) glioma. Kaplan–Meier analysis and log-rank test were performed to compare the overall survival of glioma patients with MRS findings. A cut-off value for the Glx/tCr ratio in overall survival analysis was determined based on values given in the literature as Glx and tCr concentrations in normal-appearing white matter (Glx/tCr = 1.5) [35]. The survival time was defined as the time length from the MRS examination date to the date of death. In addition, Spearman correlation analysis was performed between metabolite/tCr ratios using R v4.4.1 in RStudio v2024.04.2 [36]. Results were considered significant at *p* < 0.05.

## Results

### Patients characteristics

Of 38 patients recruited for the clinical trial, 35 patients were examined with the complete SVS MRS protocol. One patient diagnosed with metastases to the CNS (adenocarcinoma) and one patient with undefined histological status were excluded from the study. Among the remaining patients diagnosed with diffuse glioma, three patients had undergone partial resection before study inclusion, one had been treated with chemotherapy (temozolomide), and one with radiation therapy (one of the patients with partial resection). All of the pretreated patients were those with IDHmut gliomas. Partial resection was performed 4, 14, and 44 months before study inclusion. Based on the data exclusion criteria accounting for spectral quality, 7 datasets were also excluded from the statistical analysis.

Tissue samples of the remaining 26 patients (age range = 27–78 years, median age = 43) were classified according to the 2021 WHO classification of tumors of the CNS [3]. Twenty tumors were IDHmut (CNS WHO grades 2 [*n* = 5], 3 [*n* = 14], 4 [*n* = 1]), and 6 tumors were determined as IDHwt (CNS WHO grades 4 [*n* = 6]).

### Spectral quality

**Fig. 1:**
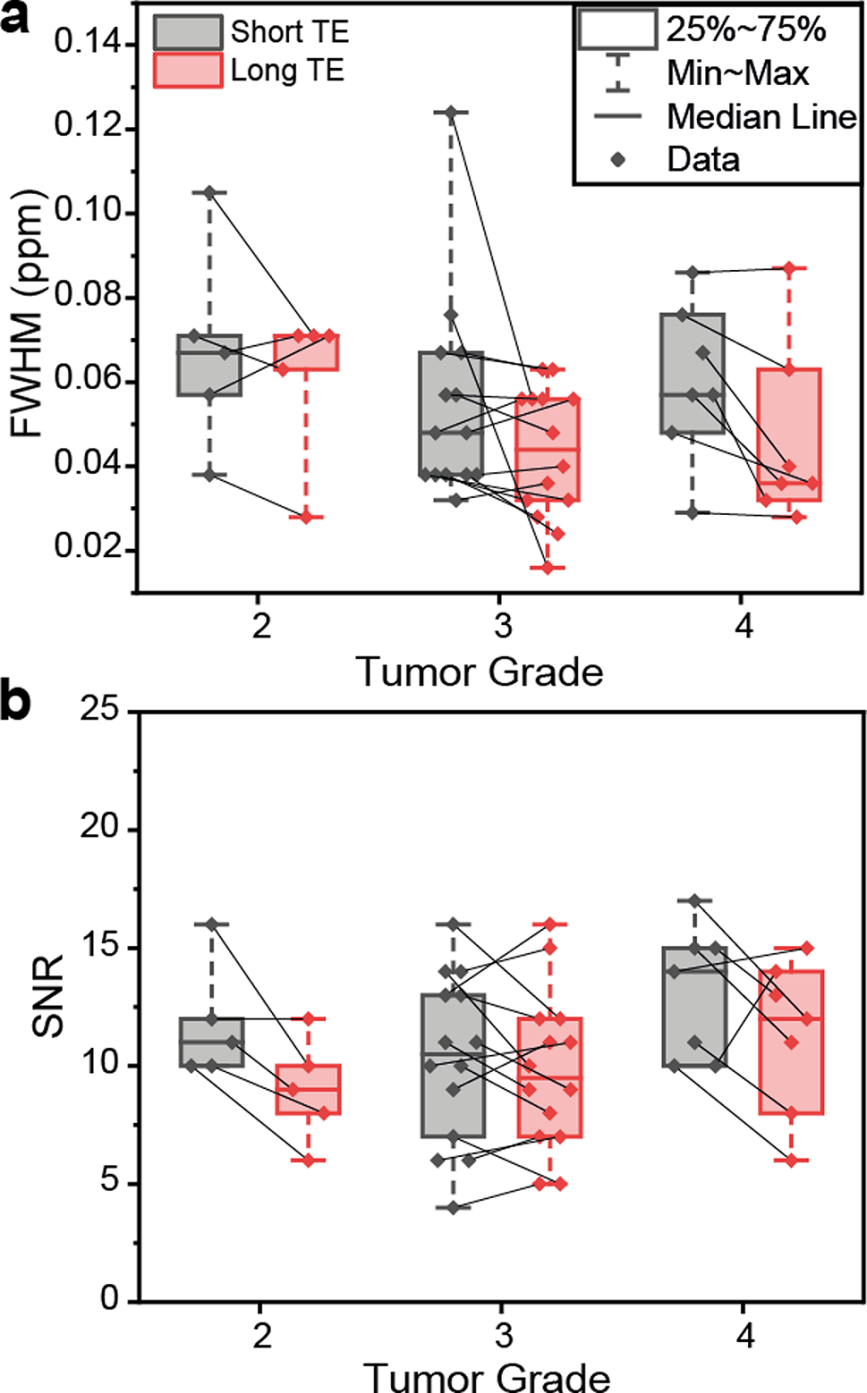
Spectral quality parameters, i.e., (**a**) full width at half maximum [FWHM (ppm)] and (**b**) signal-to-noise ratio (SNR). Each data point acquired with a short TE is connected with a corresponding data point acquired with a long TE

We defined a detection limit based on CLRBs for discriminating Ala, Gly, and mIns concentrations between the different groups. For this approach a consistent spectral quality is crucial. Spectral quality parameters, i.e., SNR and FWHM were analyzed for subgroups (Fig. 1). Line widths of two datasets acquired at short TE exceeded the exclusion limit while the linewidth of corresponding spectra obtained at long TE were within the limits. Those two spectra with broader peaks were excluded from the statistical analysis. No significant difference was observed in spectral quality parameters between different tumor subgroups.

### Alanine detection in glioma

**Fig. 2:**
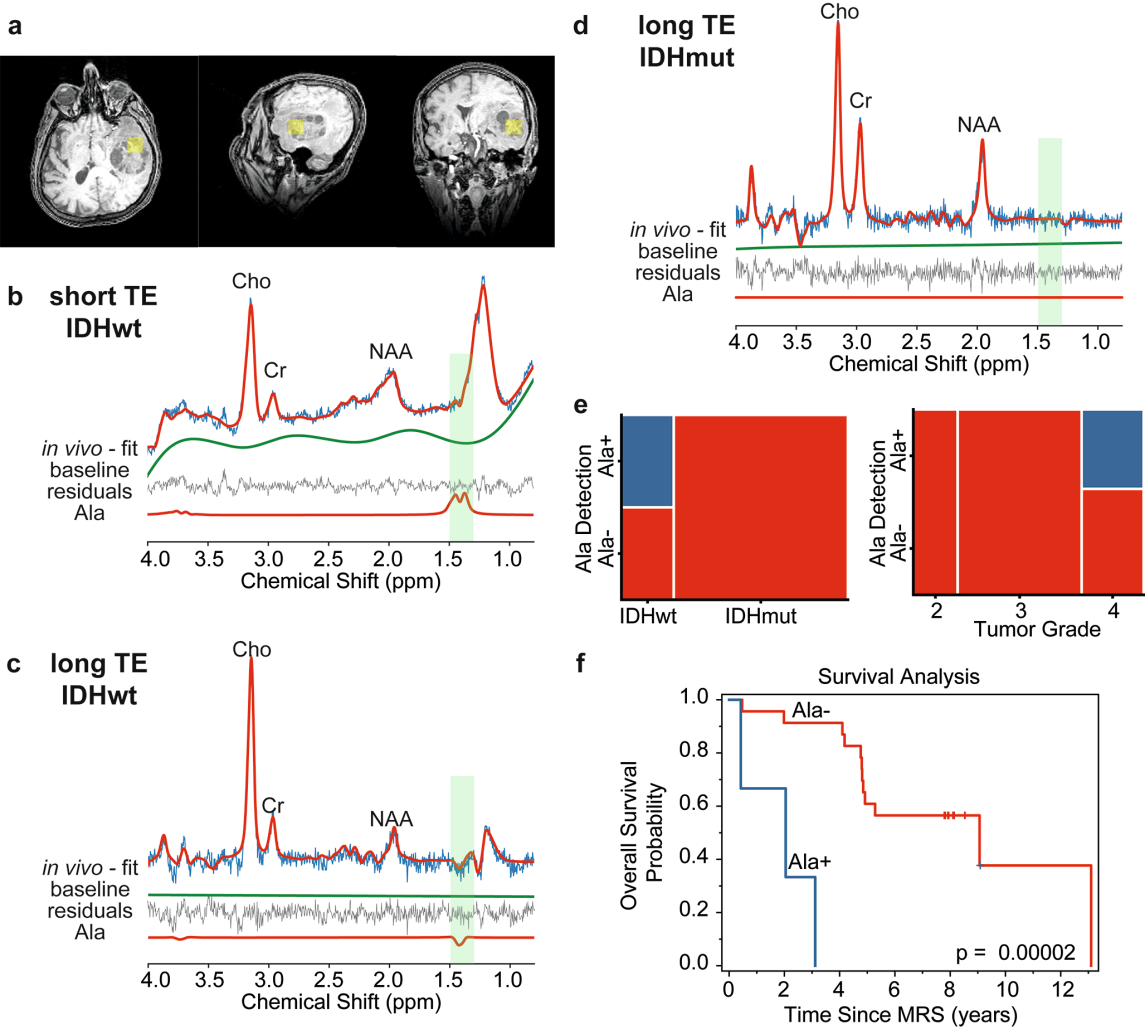
Example spectra of IDHwt glioma acquired with (**a**) voxel positioning (yellow box) shown on T1WI using (**b**) short and (**c**) long TE in addition to (**d**) an example long-TE spectrum of IDHmut glioma. (**e**) Mosaic plots demonstrating the relationship between the detection of alanine (Ala) metabolite in long-TE spectra with IDH mutation status and tumor grade, respectively. (**f**) Kaplan–Meier survival curve for overall survival stratified into groups by Ala detection in MRS examination

As shown in Fig. 2b for an example case, the Ala doublet signal appearing at 1.3–1.5 ppm range in the short-TE spectrum substantially overlaps with a large lipid signal at around 1-1.4 ppm, preventing a reliable Ala quantification. At the long TE (Fig. 2c), the Ala peak appears inverted as a result of J-coupling evolution. This inverted signal is clearly visible since the signals of lipids and macromolecules are also partially suppressed due to their shorter T2 relaxation times. In this study, Ala was detected only in IDHwt (*p* = 0.0008) and WHO grade 4 tumors (*p* = 0.01) as demonstrated with mosaic plots in Fig. 2e. On the other hand, Ala/tCr ratio was not significantly correlated with 2-HG/tCr ratio (Supp Fig. 2). Kaplan–Meier analysis (Fig. 2f) showed that overall survival was significantly shorter in glioma patients with Ala detection (Ala/tCr = 0.29 ± 0.11, 3 cases) compared to those with no Ala detection (fitting uncertainty > 40%, 23 cases) (*p* = 0.00002).

### Glutamate and glutamine (Glx) levels in glioma

Glx was detected in all cases with CRLB < 15%. There was no significant difference in the Glx/tCr ratio between IDHmut and IDHwt tumors (1.69 ± 0.78 to 2.43 ± 0.84, *p* = 0.09). On the other hand, the Glx/tCr ratio was significantly higher in WHO grade 4 (2.73 ± 1.11) compared to WHO grade 2 (to 1.28 ± 0.27, *p* = 0.005) and WHO grade 3 (to 1.60 ± 0.31, *p* = 0.003). Overall survival was not significantly different in glioma patients with Glx/tCr ratio of higher than 1.5 compared (15 cases) to those with less than 1.5 (10 cases) (*p* = 0.13).

### Glycine detection in glioma

**Fig. 3:**
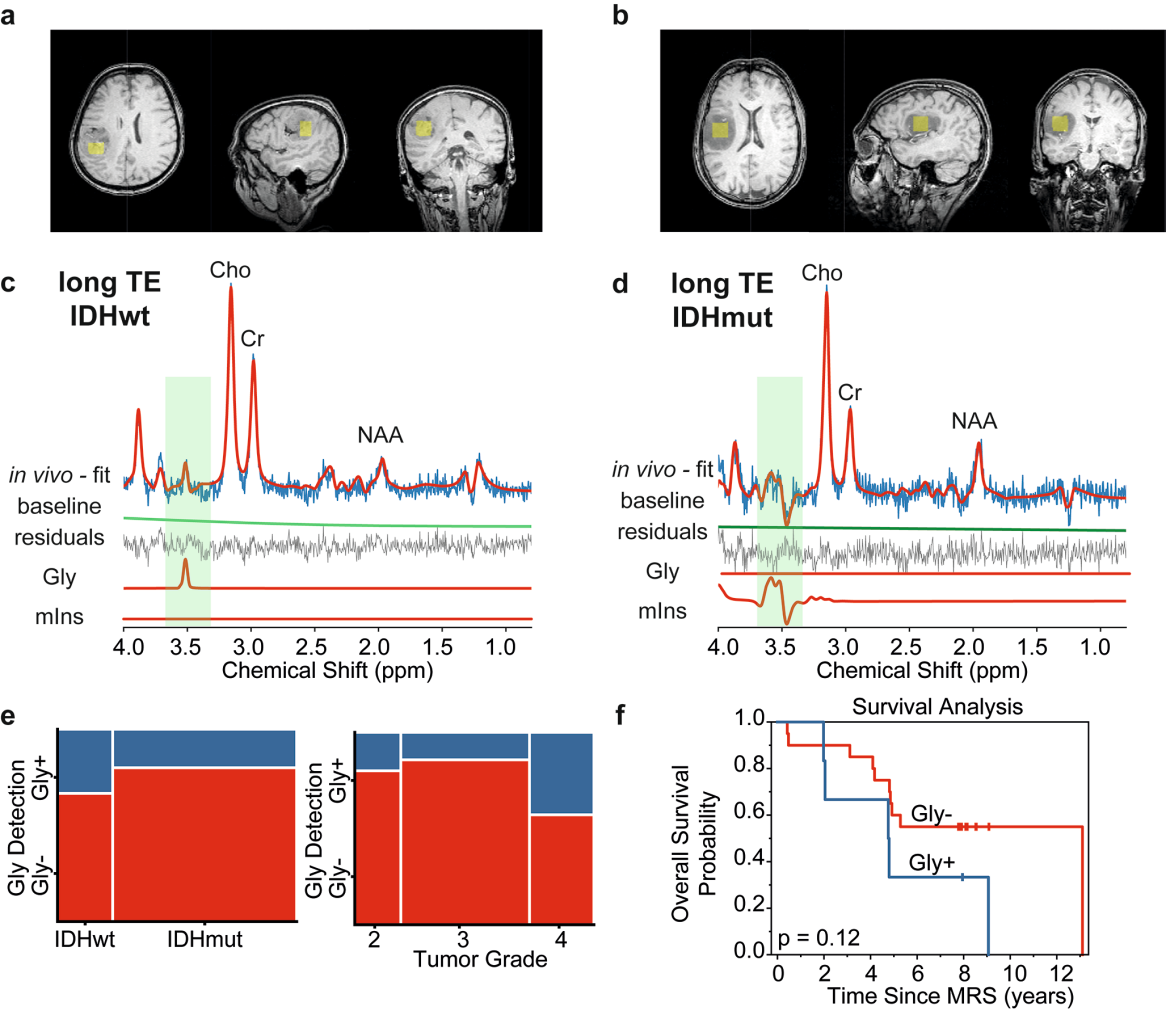
Glycine (Gly) detectability in glioma using long-TE PRESS. (**a**, **b**) Yellow boxes on T1WI indicate MRS voxel positioning for two example datasets from (**c**) IDHwt and (**d**) IDHmut glioma. Respective myo-inositol (mIns) and Gly fitting are demonstrated below. Distinct patterns of Gly and mIns are highlighted with green boxes. (**e**) Mosaic plots illustrate the relationship between the detection of Gly in long-TE spectra with IDH mutation status and tumor grade, respectively. (**f**) Kaplan–Meier survival curve for overall survival stratified into groups by Gly detection in MRS examination

As shown in Fig. 3 for two example cases, Gly and mIns appear with distinct patterns at long-TE. In the example spectral fitting presented in Fig. 3c, Gly was detected reliably (with 10% CRLB), while mIns peaks were not evident. On the other hand, in the spectrum presented in Fig. 3d, mIns multiplets were well-fitted with 7% CRLB, while Gly was not detected (see the material and methods section for a detailed explanation).

Gly detectability in long-TE tumor spectra was not significantly different between IDH mutation status (*p* = 0.50), as well as tumor grades (*p* = 0.34) (Fig. 3e).

Kaplan–Meier analysis (Fig. 3f) showed that overall survival was not significantly different in glioma patients with Gly detection (Gly/tCr = 0.12 ± 0.04, 6 cases) compared to those with no Gly detection (fitting uncertainty > 40%, 20 cases) (*p* = 0.12).

### Myo-inositol detection in glioma

**Fig. 4:**
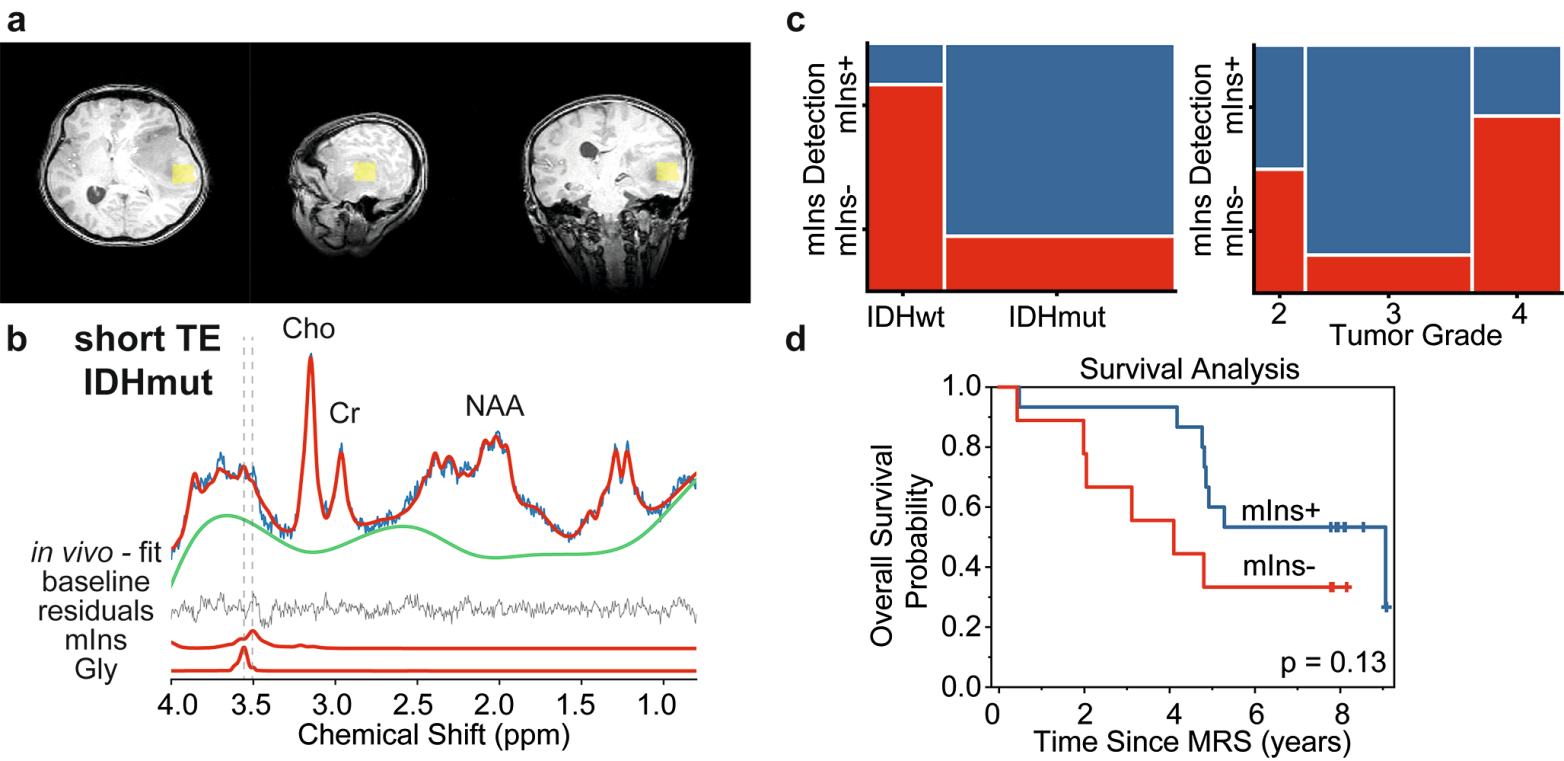
Myo-inositol (mIns) detectability in glioma with short TE PRESS. (**a**) Yellow boxes on T1WI show an example of voxel positioning for IDHmut glioma with WHO Grade 4. (**b**) Example demonstration of mIns and glycine (Gly) spectral fitting for a dataset acquired with short TE. (**c**) Mosaic plots demonstrate the relationship between the detection of mIns in short-TE spectra with IDH mutation status and tumor grade, respectively. (**d**) Kaplan–Meier survival curve for overall survival stratified into groups by mIns detection in MRS examination

As shown for an example case in Fig. 4a, b, the multiplet signal of mIns overlaps with the Gly singlet peak at 3.55 ppm challenging the spectral analysis. The detectability of mIns at short-TE spectra was significantly lower in IDHwt compared to IDHmut tumors (*p* = 0.007, Fig. 4c). Significantly decreased mIns detectability was also observed for WHO grade 4 tumors compared to WHO grade 2 and 3 tumors (*p* = 0.04, Fig. 4c).

Since the existence of Gly in the basis-set reduces the mIns intensity and increases CRLBs, we analyzed short-TE spectra also without Gly in the basis-set (Supp Fig. 3a). As an outcome of this analysis, mIns was not detected in IDHwt and WHO grade 4 tumor groups only (Supp Fig. 3b, c). These detectability differences between tumor groups were significant with *p*-values of 0.0002 and 0.003, respectively.

Kaplan–Meier analysis (Fig. 4d) showed that overall survival was not significantly different in glioma patients with detection of mIns (mIns/tCr = 0.60 ± 0.35, 15 cases) compared to those with no mIns detection (fitting uncertainty > 40%, 9 cases) (*p* = 0.13).

## Discussion

In this study, we investigated the association between the MRS-detectable brain amino acids (Ala, Gly, Glu, and Gln) as well as mIns and glioma-defining molecular features (i.e., IDH mutation status), CNS WHO grade, and patient overall survival. In vivo single-voxel spectra from tumor tissue were recorded on a clinical MR scanner at short and long TE. Due to the inherent problems of low metabolite concentrations and overlapping signals in MRS at field strength ≤ 3 T, we resorted to a rather conservative approach relying on binary categories for metabolite concentrations with detectable/non-detectable in tumor tissue for Ala, Gly, and mIns. The use of such an approach can cause errors in the analysis as a result of spectral quality differences between tumor groups. Nevertheless, we included only high-quality spectral data in the analysis and observed no relevant difference in spectral quality parameters between the groups.

Our main finding was a strong association between the intratumoral Ala detection (i.e., higher Ala concentration) and the glioma-defining molecular feature IDHwt as well as CNS WHO grade 4. In addition, the overall survival of glioma patients with Ala detection in vivo MR spectra was considerably shorter than that of patients without Ala detection (lower Ala concentration) which was an expected finding due to the association of Ala detection with IDHwt status. Using untargeted NMR-based metabolomics and machine learning, Firdous et al. identified Ala as a key metabolite for discriminating between glioma and non-glioma tissue [18]. In addition, Pearl et al. measured tumor Ala levels using ex vivo HR-MAS NMR and reported significantly lower Ala levels in IDHmut glioma tissue compared to IDHwt tissue. Further, they demonstrated that the Ala concentration varies significantly as a function of survival status, which corroborates our findings [37]. Ala is involved in the brain’s ammonia transfer. Ala aminotransferase transfers an amino group from Ala to α-ketoglutarate, resulting in the production of Glu and pyruvate [38]. Pyruvate enters the metabolic mainstream to supply energy and nutrients for rapidly proliferating tumor cells [39]. While this mechanism might explain the relationship between high Ala concentrations and a poor prognosis, the dysregulation in the Glu pathways might be an explanation for low Ala levels in IDHmut tumors [37].

We found a higher Glx/tCr ratio in WHO grade 4 compared to 2 and 3 tumors. In literature, a higher concentration of Glx in high-grade compared to low-grade gliomas has been reported by Chawla et al. where MR perfusion imaging (arterial spin labeling) was used to guide the spectral measurement location [40]. Extracellular Glu levels in the glioma microenvironment are up to 100 times higher than in normal brain tissue [41–43]. These high levels of Glu stimulate glioma cell proliferation and invasion [44–46] and may lead to glioma-associated epileptic discharges and excitotoxicity [47, 48]. Gln is a major substrate to produce Glu. Tumor cells can be addicted to Gln because it is a nitrogen reservoir required for cell proliferation [49]. Since an increase in Gln concentration in tumor cells is correlated with tumor growth, enzymes involved in the glutaminolysis pathway have become a target for pharmacological therapy strategies [50]. As reported previously, in our patient group, the Glx/tCr ratio did not discriminate IDHwt from IDHmut [29]. We observed no association between the patients’ overall survival and the Glx/tCr ratio while Sacli-Bilmez et al. reported an association between high Glx/tCr level (> 2.966 fold) and poor overall survival in the group of patients with IDHwt, TERTp mutation gliomas [51]. Quantification of Gln and Glu together as Glx could conceal changes in Gln by concomitant alterations in Glu levels. This limitation might be addressed using specific MRS sequences and ultrahigh-field MRS quantifying Glu and Gln separately [52, 53].

We found no association between Gly detectability in tumor tissue and IDH mutation status or tumor grade. However, we observed a relevant difference in Gly concentration in high-grade gliomas compared to low-grade gliomas in our previous clinical study with MRS [54]. The discrepancy between the findings of the studies might originate from the use of different editions of the WHO classification of tumors of the central nervous system (third vs. sixth editions) [3, 23]. In addition, at the time common grouping of low-grade (WHO grades I and II) and high-grade (WHO grades III and IV), based on the tumors’ growth potential and aggressiveness, was not precise and is now outdated. Consistent with our results, Tiwari et al. reported no difference in Gly concentration between IDHwt and IDHmut groups. Additionally, they demonstrated an association between high Gly concentration (2.5 mM) and shorter overall survival [12], which we observed as a trend that did not reach significance. This may be attributed to the limitations of the binary approach, which assigns the detectability of Gly only to a rather small fraction of the examined tumors and ultra-high field MRS might be beneficial to elucidate the role of this metabolite in tumor metabolism [53, 55].

Due to its overlapping signals with the Gly peak in spectra acquired at ≤ 3 T and its known diagnostic value in glioma, we additionally analyzed mIns detectability [21]. mIns is defined as a glial marker and high mIns levels are associated with glial proliferation [56]. Short-TE is preferential for mIns detection to not suffer from T2-decay and dephasing resulting in low signal intensity and hence low spectral fitting precision [21]. As demonstrated here, the addition of a Gly peak to the basis set affects the calculated mIns level noticeably due to variations in mIns spectral fitting. This is particularly important in glioma where an increase in Gly concentration may be observed as reported above. Another approach is to quantify the sum of mIns and Gly which might cause concealing the individual alterations in Gly and mIns levels in glioma.

In this study, the non-detectability of mIns (i.e., low mIns level) was associated with IDHwt and WHO grade 4 tumors, regardless of the inclusion of the Gly signal in the basis set. In consistence with these findings, Castillo et al. reported higher mIns/Cr levels in low-grade tumors compared to anaplastic astrocytoma and glioblastoma [57] and Hangel et al. found lower mIns levels to be associated with higher grade glioma in a 7 T MRSI study, where separate quantification of mIns and Gly is improved [53]. This pattern may be explained by the lower availability of the glial marker in high-grade compared to low-grade glioma.

With regard to the IDH mutation status, our findings were corroborated with the study by Bumes et al. where lower mIns levels were observed in IDHwt tumors compared to IDHmut [58]. However, despite the association between the mIns detectability and IDHwt along with high-grade tumors, we observed no association between its detectability and patients’ overall survival– such as strongly observed for the Ala detection. This might point towards a strong relationship between Ala detection in tumors and poor overall survival, independent of the confounding effects of IDH mutation status and tumor grade.

### Limitations

The study is limited by sample imbalance between classes. The low signal intensity of amino acids led us to perform binary quantification which might lower the power of statistical analysis. Our approach of defining detection limits on the basis of CRLBs < 40% establishes a threshold that depends on the accuracy of the method and is therefore arbitrary. Improving the method might shift this threshold to even lower values. However, with regard to Gly and Ala, which cannot be detected in normal-appearing brain tissue with conventional MRS techniques, the approach is reasonable since it discriminates tumors with significant changes of the respective amino acids from others and, for Ala, reveals a significant difference in outcome for those tumors. As mIns is detectable in normal-appearing brain tissue, we aimed at tumors with rather low (i.e. non-detectable) mIns levels. Our results indicate the significance of non-detectable as threshold, although higher accuracy might help in fine-tuning this parameter. In the case of Glx, which can be quantified with high fitting precision, metabolite ratio (/tCr) was calculated due to the lack of water quantification in the protocol. Tumor-specific voxel positioning in single-voxel MRS is an additional significant factor that might influence the outcome due to intratumoral heterogeneity.

## Conclusion

Focusing on amino acids in MRS can improve its diagnostic and prognostic value in glioma. Ala, which is visible at long TE even in the presence of lipids, could be a relevant indicator for overall survival.

## Electronic supplementary material

Below is the link to the electronic supplementary material.

Supplementary Material 1

## Data Availability

Datasets generated during the current study are available from the corresponding author on reasonable request.
