# Supplementary material for: Amino acid metabolism in glioma: in vivo MR-spectroscopic detection of alanine as a potential biomarker of poor survival in glioma patients
Source: J Neurooncol. 2024 Aug 27;170(2):451–61. doi: 10.1007/s11060-024-04803-2 (PMC11538230; doi:10.1007/s11060-024-04803-2)
Supplement: Supplementary file 1 — Supplementary Material 1 [file 11060_2024_4803_MOESM1_ESM.pdf]

Supplementary Information: Amino Acid Metabolism in Glioma: *In vivo* MR-spectroscopic Detection of Alanine as a Potential Biomarker of Poor Survival in Glioma Patients

CRLB Values of Low-concentrated Metabolite Spectral Fitting in Glioma

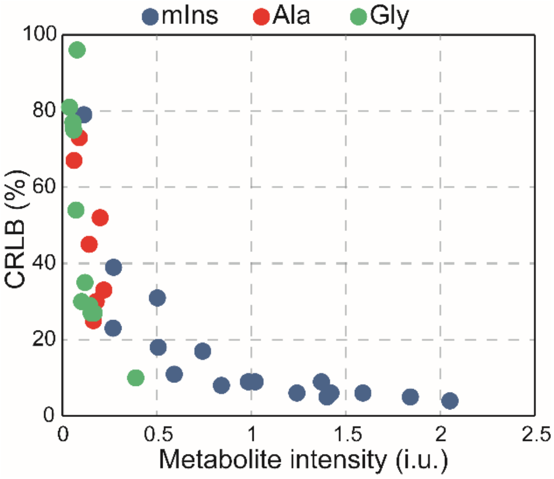

Supplementary Figure 1. Relative Cramer-Rao lower bound (CRLB) values versus metabolite intensities for myo-inositol (mIns, blue circle), alanine (Ala, red circle), and glycine (Gly, green circle) given by LCModel. Only CRLB values <100% and metabolite intensity >0 are shown here.

Correlation between metabolite/Cr ratios

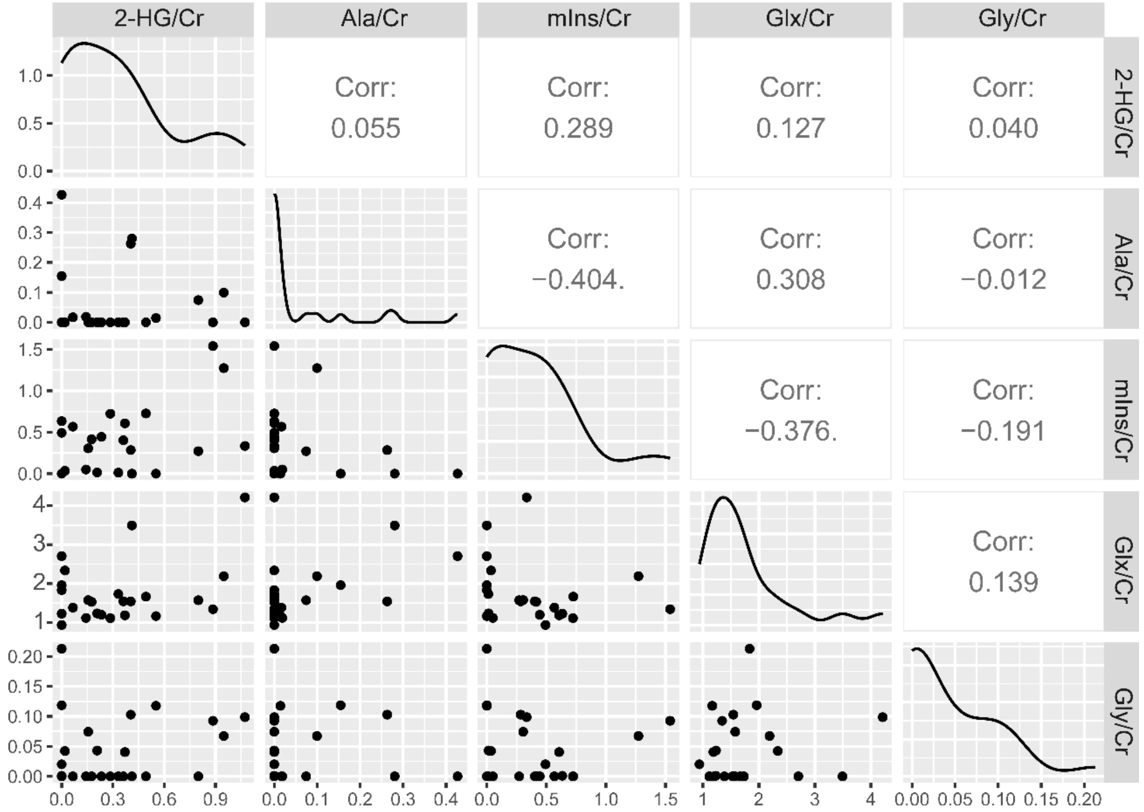

Supplementary Figure 2. Spearman correlation plot for 2-HG, aminoacid (i.e., Gly, Ala, Glx) and mIns to Cr ratios. Scatter plots in the lower triangle show pairwise relationships between metabolites of interest. Spearman correlation coefficients are given in the upper triangle. The diagonal presents density plots for each metabolite to illustrate their distributions.

mIns Detection in Glioma

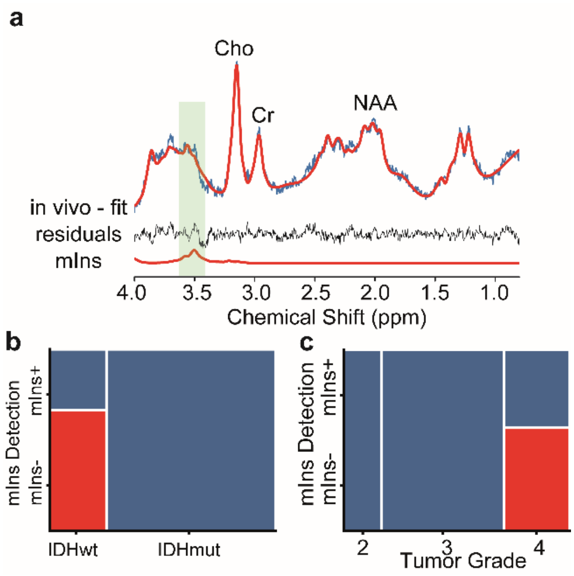

Supplementary Figure 3. (a) The data was evaluated without glycine (Glyc) spectral simulation in the basis set. Mosaic plots demonstrating the relationship between the detection of myoinositol (mIns) in short TE spectra with (b) IDH mutation and (c) tumor grade where the spectral analysis is performed without Glyc fitting.

MRSinMRS checklist

The Minimum Reporting Standards for in vivo Magnetic Resonance Spectroscopy (MRSinMRS) checklist can be found in **Supplementary Table 1.**

Supplementary Table 1. MRSinMRS checklist for our multi-sequence MRS protocol.

Site (Name or Number)		
1. Hardware		
a. Field strength [T]	3 T	3 T
b. Manufacturer	Siemens	Siemens
c. Model (software version if available)	Trio (syngo MR A35)	Trio (syngo MR A35)
d. RF coils: nuclei (transmit/ receive), number of channels, type, body part	double-tuned $^{1}H/^{31}P$ volume head coil	double-tuned $^{1}H/^{31}P$ volume head coil
e. Additional hardware	N/A	N/A

2. Acquisition		
a. Pulse sequence	^{1}H PRESS SVS	^{1}H PRESS SVS
b. Volume of Interest (VOI) locations	Patients: tumor	Patients: tumor
c. Nominal VOI size [cm^3, mm^3]	20 x 20 x 20 mm^3	20 x 20 x 20 mm^3
d. Repetition Time (TR), Echo Time (TE) [ms, s]	TR = 3000 ms, TE = 30 ms	TR = 3000 ms, TE = 97 ms
e. Total number of Excitations or acquisitions per spectrum<br>In time series for kinetic studies<br>i. Number of Averaged spectra (NA) per time-point<br>ii. Averaging method (e.g. block-wise or moving average)<br>iii. Total number of spectra (acquired / in time-series)	96	128
f. Additional sequence parameters<br>(spectral width in Hz, number of spectral points, frequency offsets)<br>If STEAM:, Mixing Time (TM)<br>If MRSI: 2D or 3D, FOV in all directions, matrix size, acceleration factors, sampling method	1200 Hz, 1024 points	1000 Hz, 1024 points<br>Sinc-shaped excitation pulse (duration<br>2.6 ms, Slice selection gradient<br>amplitude 33.95 mT/m, BWTP 8.75),<br>Mao refocusing pulse (duration,<br>2.6 ms, section-refocusing gradient<br>amplitude, 2.7171 mT/m; BWTP 6)
g. Water Suppression Method	CHESS	CHESS
h. Shimming Method, reference peak, and thresholds for	Automated 3D B0 field mapping technique followed by manual adjustment <25 Hz	Automated 3D B0 field mapping technique followed by manual adjustment <25 Hz

“acceptance of shim” chosen		
i. Triggering or motion correction method<br><br>(respiratory, peripheral, cardiac triggering, incl. device used and delays)	N/A	N/A
3. Data analysis methods and outputs		
a. Analysis software	LCmodel 6.3	LCmodel 6.3
b. Processing steps deviating from quoted reference or product	Basis set created using jMRUI 5.2 plug-in NMR-ScopeB	Basis set created using jMRUI 5.2 plug-in NMR-ScopeB
c. Output measure<br><br>(e.g. absolute concentration, institutional units, ratio)Processing steps deviating from quoted reference or product	Ratio to creatine and binary quantification for Ala, mIns, and Gly with categories detectable/non-detectable based on CRLB threshold (40%) as explained in the manuscript	Ratio to creatine and binary quantification for Ala, mIns, and Gly with categories detectable/non-detectable based on CRLB threshold (40%) as explained in the manuscript
d. Quantification references and assumptions, fitting model assumptions	2-hydroxyglutarate, N-acetylaspartate, N-acetylaspartyl glutamate, choline, creatine, glutamate, glutamine, myo-inositol, alanine, and lactate simulated using real pulses. Gly singlet peak simulation implemented in LCModel analysis. Macromolecules were not modeled.	2-hydroxyglutarate, N-acetylaspartate, N-acetylaspartyl glutamate, choline, creatine, glutamate, glutamine, myo-inositol, alanine, and lactate simulated using real pulses. Gly singlet peak simulation implemented in LCModel analysis. Macromolecules were not modeled.
4. Data Quality		
a. Reported variables<br><br>(SNR, Linewidth (with reference peaks))	SNR and line widths are presented in Figure 1.	SNR and line widths are presented in Figure 1.
b. Data exclusion criteria	existing artifacts, metabolite linewidth (FWHM) > 0.1 ppm, and signal-to-noise ratio < 3	existing artifacts, metabolite linewidth (FWHM) > 0.1 ppm, and signal-to-noise ratio < 3
c. Quality measures of postprocessing Model	Rejection thresholds using Cramer-Rao lower	Rejection thresholds using Cramer-Rao lower

fitting (e.g. CRLB, goodness of fit, SD of residual)	bounds (CRLBs) of metabolite fits were defined as CRLB <10% for total Cho and total Cr (tCr), <15% for Glu+Gln (Glx). There was no data rejected due to the CRLB threshold.	bounds (CRLBs) of metabolite fits were defined as CRLB <10% for total Cho and total Cr (tCr), <15% for Glu+Gln (Glx). There was no data rejected due to the CRLB threshold.
d. Sample Spectrum	Figure 2-4 and Supplementary Figure 2	Figure 2-4 and Supplementary Figure 2
